# Polysaccharides and oligosaccharides originated from green algae: structure, extraction, purification, activity and applications

**DOI:** 10.1186/s40643-024-00800-5

**Published:** 2024-09-06

**Authors:** Chen Li, Hui Wang, Benwei Zhu, Zhong Yao, Limin Ning

**Affiliations:** 1 College of Food Science and Light Industry, Nanjing Tech University, Nanjing, 211816 China; 2 College of Medicine, Nanjing University of Chinese Medicine, Nanjing, 210023 China

**Keywords:** Green algae polysaccharide, Oligosaccharide, Structure, Extraction, Activity

## Abstract

**Abstract:**

With the proceeding of global warming and water eutrophication, the phenomenon of green tide has garnered significant societal interest. Consequently, researchers had increasingly focused on the potential applications of green algae biomass, particularly its polysaccharides. The polysaccharide serves as the primary active constituent of green algae and has demonstrated numerous advantageous biological activities, including antioxidant, antiviral, anticoagulant, hypolipidemic and immuno-modulatory activities. The favorable bioavailability and solubility of green algae oligosaccharides are attributed to their low molecular weight. So there has been a growing interest in researching green algae polysaccharides and oligosaccharides for the utilization of marine biological resources. This review summarized the extraction, purification, chemical structure, composition, biological activity, and potential applications prospect of polysaccharides and oligosaccharides derived from green algae. The review could be helpful for expanding the applications of polysaccharides and oligosaccharides of green algae.

**Graphical Abstract:**

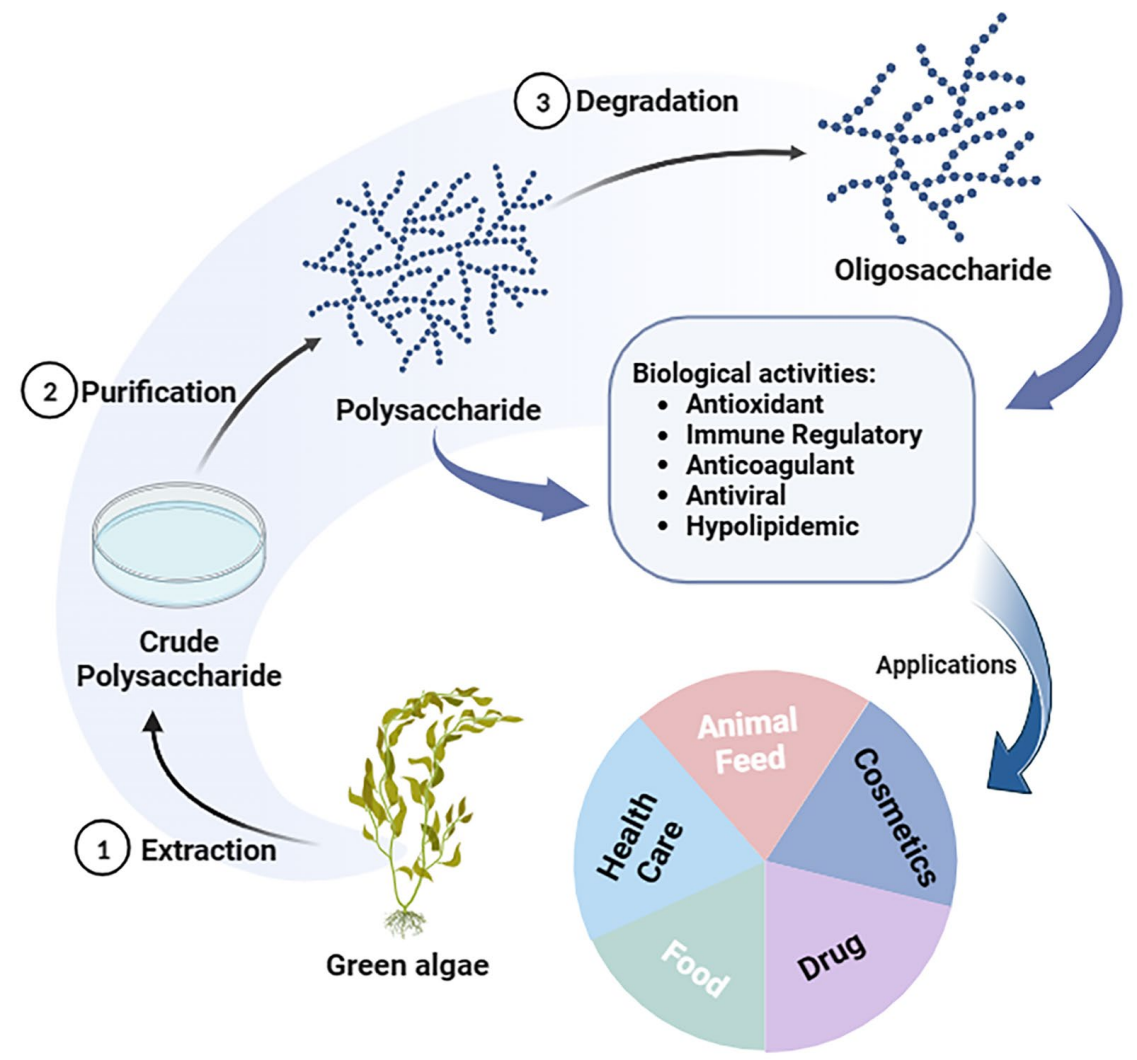

## Introduction

**Fig. 1:**
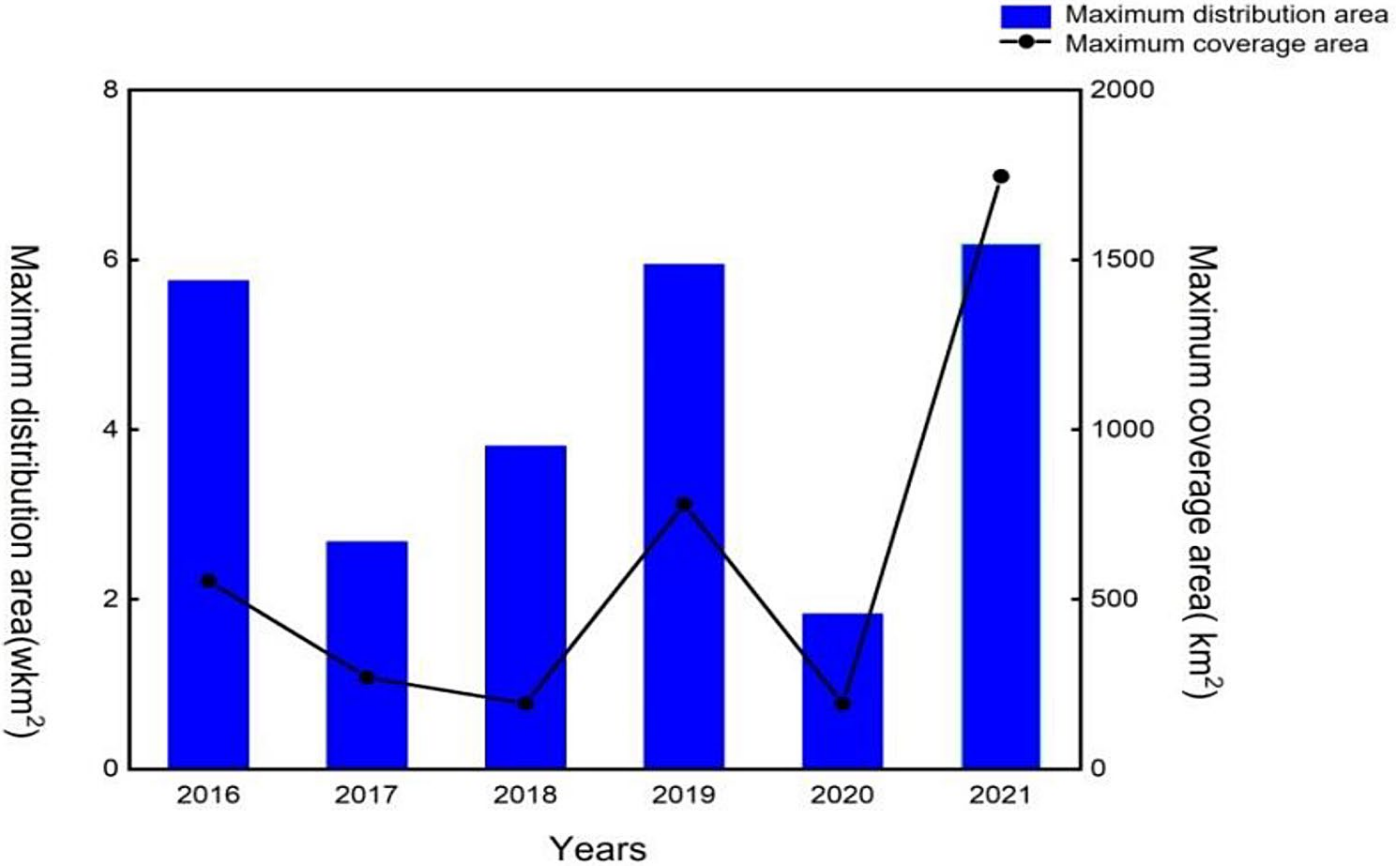
Changes of green tide distribution area and coverage area in the Yellow Sea in recent years

With over 70% of the Earth’s surface being covered by oceans and approximately half of total global biodiversity consisting of marine species, marine organisms serve as valuable sources of natural products possessing diverse biological activities and structures (Aneiros et al. 2004, Kim et al. 2010, Wijesekara et al. 2011). Common algae found in the sea include brown algae, red algae, cyanobacteria, and green algae, many of which are edible and offer high nutritional value. Examples of edible algae include *Laminaria japonica* (brown algae), *Gelidium amansii Lamouroux* and *Chondrus ocellatus Aolmes* (red algae) (Wijesekara et al. 2011), and *Ulva* and *Enteromorpha* (green algae). Brown and red algae, specifically carrageenan and alginate, are extensively utilized, whereas research on green algae has been comparatively limited. In recent years, occurrences of “green tides” along coastlines have been frequently observed, primarily attributed to the proliferation of *Ulva* and *Enteromorpha* (Li et al. 2016b, c). This ecological anomaly is characterized by the rapid growth or aggregation of large green algae, detached from their original substrate, forming floating clusters. If not treated in time, the consequences of this issue include the mortality of marine animals, depletion of oxygen in algae and seawater, and deterioration of seagrass habitats. Additionally, it will have significant impacts on the economic and ecological well-being of coastal cities (Fan et al. 2022). Against the backdrop of offshore eutrophication and global warming, the escalation of green tide occurrences is of great concern (Smetacek et al. 2013, Van Alstyne et al. 2015; Wang et al. 2015; Zhang et al. 2015; Coelho et al. 2016, Gao et al. 2017) (Fig. 1). Therefore, there is an urgent need to dig deep into the biological resource potential of green algae and effectively harness these resources.

**Fig. 2:**
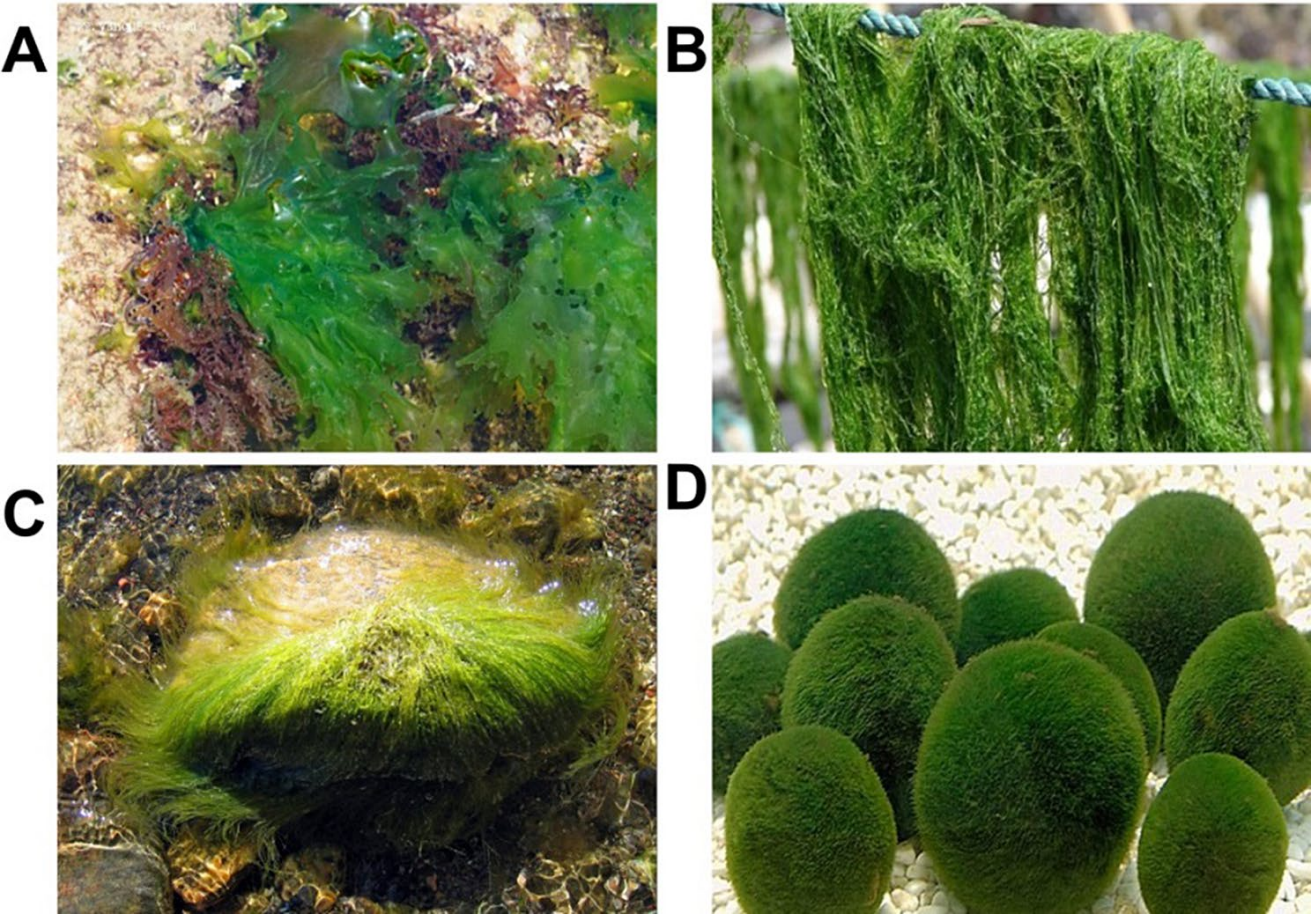
The morphology of some representative species of green algae. (**A**). Ulva lactuca; (**B**). Enteromorpha prolifera; (**C**). Monostroma nitidum; (**D**). Chlorella vulgaris

**Fig. 3:**
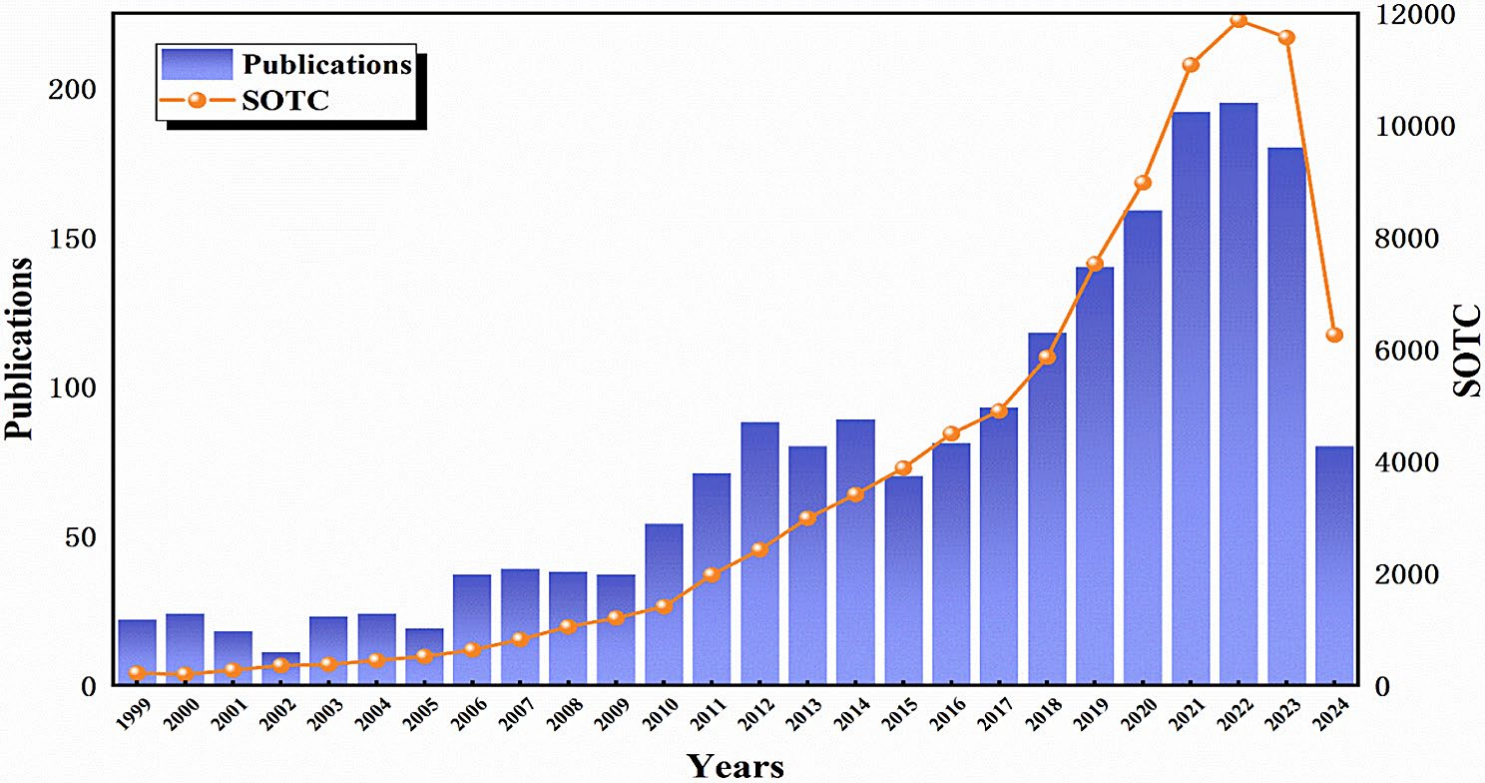
Number of publications and SOTC on green algae polysaccharides from 1999

At present, brown algae and red algae have been extensively cultivated and industrialized among the four major types of seaweed. In contrast, green algae, despite being the most diverse type, has not been widely exploited and utilized, with only select high-yield varieties being utilized for purposes such as feed, bait, and fertilizer. Green algae are rich in nutritional value, boasting low fat content and abundant protein, cellulose, and trace elements. Additionally, it contains over ten types of polyunsaturated fatty acids, including linoleic acid, linolenic acid, palmitic acid, and arachidonic acid. As research on green algae continues to advance, its utilization across various industries, including industry, agriculture, medicine, and food, has become increasingly prevalent (Muhamad et al. 2019; Rial-Hermida et al. 2021; Srivastava et al. 2021; Vinchhi et al. 2021) (Fig. 2). Green algae, serving as a valuable source of raw materials for the development of feed, food, and pharmaceutical products, are primarily composed of green algae polysaccharides. The green algae polysaccharides have garnered significant attention due to their biocompatibility, low toxicity, mini-mal side effects, natural residue free properties, non-polluting nature, and lack of drug resistance (Wassie et al. 2021). A comprehensive search of the Web of Science database (www.webofscience.com, accessed on 31 July 2024) utilizing the keyword “green algae polysaccharides” resulted in the identification of 2,179 publications. Notably, the majority of these publications were released post-2010 (Fig. 3). Green algae polysaccharides are abundant in green algae, exhibiting diverse components and structures that contribute to various biological activities such as anti-coagulation, anti-viral, immune regulation, lipid-lowering, anti-radiation, anti-oxidation, and anti-tumor properties (Abd-Ellatef et al. 2017; Fournière et al. 2019; Kidgell et al. 2019; Chi et al. 2020b; Jiang et al. 2020; Klongklaew et al. 2020; Ponce et al. 2020). The green algae oligosaccharides, derived from the degradation of polysaccharides, not only retain the biological activities of polysaccharides but also enhances solubility and bioavailability. Currently, *Ulva* and *Enteromorpha* are the primary species of green algae that have been extensively studied for their polysaccharides. In recent years, there has been a growing interest among scholars in the study of green algae polysaccharides and oligosaccharides. This review examines the chemical composition, structure, separation and purification methods, and biological activities of green algae polysaccharides and oligosaccharides in nature.

## Green algae polysaccharide

### Chemical composition

Green algae polysaccharides are predominantly found in the cell interstitium and cell wall (Bobin-Dubigeon et al. 1997), primarily consisting of water-soluble sulfated polysaccharides. The chemical composition of green algae polysaccharides is influenced by the species of green algae utilized for extraction, the environmental conditions in which the algae are grown, and the time of harvest, resulting in a complex composition (Li et al. 2017; Zhong et al. 2020). Ulvan is a polysaccharide derived from green algae, predominantly consisting of glucuronic acid (GlcA), iduronic acid (IdoA), 3-sulfated rhamnose (Rha3S), and minor amounts of xylose (Xyl) (Ulaganathan et al. 2017). It exhibits low concentrations of mannose, galactose, and arabinose (Guidara et al. 2019; Jmel et al. 2019). Kidgella et al. (Kidgell et al. 2021) conducted an analysis of the monosaccharide compositions of different sources of *Ulva* and observed significant differences in the polysaccharides derived from leafy and filamentous *Ulva*. Specifically, the polysaccharides from these sources were found to be primarily composed of rhamnose, with glucuronic acid also present. Notably, the proportion of iduronic acid in the polysaccharides of filamentous *Ulva* was generally lower (approximately 7 mol%) compared to leafy *Ulva* (approximately 14 mol%). In addition to this, the polysaccharides of *U. ralfsii* exhibited a galactose proportion of 16 mol%, a significantly higher value compared to other *Ulva* species. The harvest time of *Ulva* also affects the chemical composition of the polysaccharides. Samarasinghea et al. (Samarasinghe et al. 2021) analyzed the monosaccharide composition of *Ulva* at different harvest times, revealing consistent main components but varying content levels. For instance, the dry matter content ratios of rhamnose, xylose, galactose, glucose and uronic acid in ulvan collected in June and August were 3.65:0.43:0.41:0.32:0.62 and 0.84:0.33:0.22:0.75:1.92, respectively. It is evident that ulvan harvested in August exhibit higher levels of glucose and uronic acid compared to those harvested in June. Moreover, the growing conditions also have an impact on the chemical composition of ulvan. Olsson et al. (Olsson et al. 2020) studied the effects of cultivation conditions, such as temperature, irradiance, pCO_2_, nitrogen, and phosphate, on the monosaccharide composition of ulvan. Their findings revealed that lower sulfate concentrations and high temperatures could promote an increase in monosaccharide content, while increased irradiance and temperature levels were associated with higher concentrations of rhamnose and iduronic acid.

*Enteromorpha*, as a natural resource, is abundant in nutrients, comprising essential components such as carbohydrates (43–51%), proteins (26–33%), fats (0.2–0.8%), total amino acids (20.26–23.32%), ash (13–14%), and iron (1.1–3.4 mg/g) (Li et al. 2018a; Zhang et al. 2019). *Enteromorpha* polysaccharides (EPs) primarily consist of water-soluble sulfate polysaccharides, comprising of rhamnose, xylose, mannose, galactose and glucose interconnected by glycosidic bonds (Tang et al. 2013; Li et al. 2017; Jiang et al. 2021). Qi et al. (Qi et al. 2012, 2013) studied the chemical composition of polysaccharides in different species of *Enteromorpha* (*E. linza*, *E. prolifera* and *E. clathrata*), revealing distinct differences in the chemical composition of these polysaccharides. Specifically, the polysaccharides of *E. linza* and *E. prolifera* were mainly composed of rhamnose, whereas those of *E. clathrata* contained primarily arabinose and galactose, along with minor quantities of rhamnose, fucose, and xylose. This indicated that significant variations in polysaccharide composition among different species of *Enteromorpha*. Ji et al. (Ji et al. 2009) analyzed the chemical composition of polysaccharides in *E. clathrata*, revealing the presence of rhamnose, glucuronic acid, and iduronic acid, but not mannose. This observation indicated a notable distinction in the polysaccharide components of *E. clathrata* during the outbreak stage compared to normal growth conditions. Additionally, Shi et al. investigated the monosaccharide composition of polysaccharides extracted from *E. clathrata* at different time points, finding consistent types of monosaccharides across samples, but differing levels of content. However, due to the complexity of their composition and structure, the conformational relationships of EPs are mostly unknown (Yu et al. 2017).

It could be seen that the chemical composition of green algae would vary with the different species of green algae, growth environment and harvest time, a phenomenon commonly observed in the analysis of algae polysaccharides. (Benslima et al. 2021).

### Structure

**Fig. 4:**
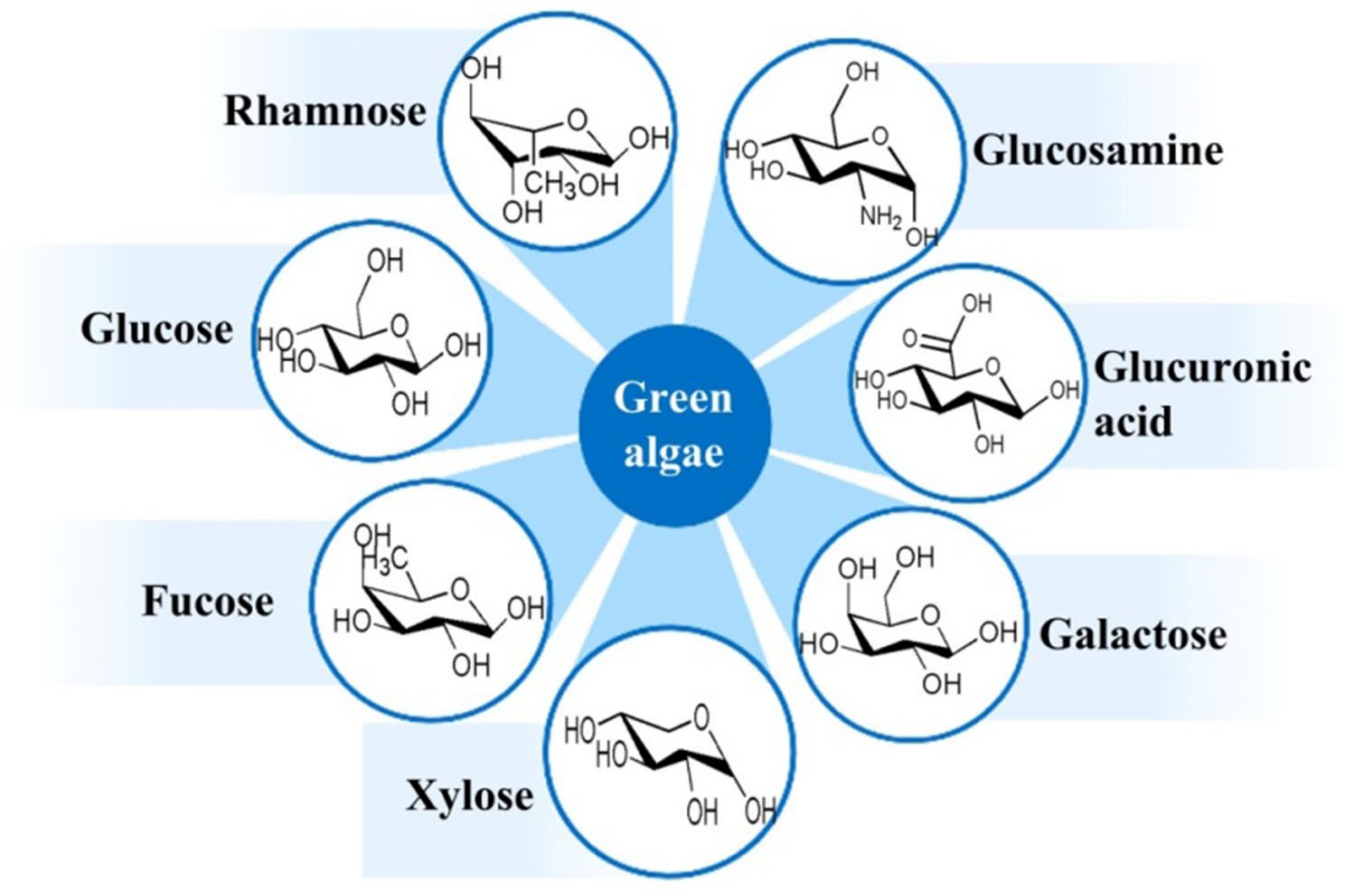
The main monosaccharide composition of green algae polysaccharides

**Fig. 5:**
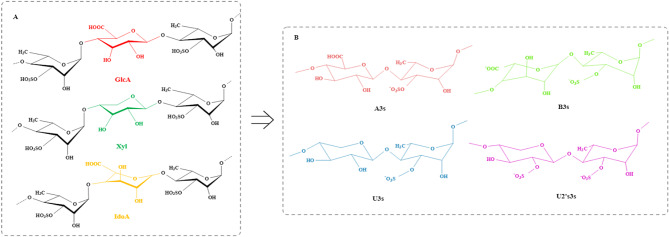
The structural characteristics (**A**) and the main disaccharide repeat units (**B**) of ulvan

The structural complexity of green algae polysaccharides surpasses that of polysaccharides found in brown and red algae, attributable to the intricate composition of monosaccharides, the diverse glycosidic linkages between these monosaccharides, and the extensive array of structural and motif modifications within their branched configurations (Yang et al. 2011; Stender et al. 2019). The structure of polysaccharides differs among various algae species (Fig. 4) (Table 1), with ulvan being the most extensively studied polysaccharide structure in green algae. Lahaye et al. (Lahaye et al. 2007) studied the structure of ulvan derived from *U. regida* and founded the main disaccharide repeat structure of ulvan was A_3s_ [→4)β-_D_-GlcA(1→4)-α-_L_-Rha3S(1→] and B_3s_ [→4)α-_L_-IdoA(1→4)-α-_L_-Rha3S(1→]. In addition, structural analysis identified the presence of U_3s_ [→4)β-_D_-Xyl(1→4)-α-_L_-Rha3S(1→] and U_2’s,3s_ [→4)β-_D_-Xyl2S(1→4)-α-_L_-Rha3S(1→] as two repeat disaccharide units. The structural characterization of ulvan can be further elucidated through the application of ulvan lyase, an endonuclease isolated from marine bacteria. Ulvan lyases catalyze the cleavage of the β-(1→4)-glycosidic bond between Rha3S and GlcA or IdoA via a β-elimination mechanism, resulting in the formation of oligosaccharides that contain unsaturated uronic acid (∆GlcA) (Collén et al. 2011; Gao et al. 2019). These oligosaccharides exhibit repeating units such as -A_3s_-A_3s_-, -A_3s_-B_3s_-, -A_3s_-U_3s_-, and -A_3s_-GlcA-A_3s_. Chi et al. (2020a) employed ulvan lyase to degrade polysaccharides derived from *U. clathrata*, resulting in three distinct degradation products with varying molecular weights, designated as UO-1, UO-2, and UO-3. Structural analysis was conducted on the higher molecular weight component, UO-3, revealing that it predominantly consists of A_3s_-type and U_3s_-type disaccharide repeating units, with the presence of U_2’s,3s_-type disaccharide repeating units also identified. It can be seen that ulvan exhibits a complex structure predominantly comprising disaccharide repeating units of the A_3s_-type or B_3s_-type, with a minor presence of U_3s_-type or U_2’s,3s_-type disaccharide repeating units, as shown in Fig. 5. The main chain of ulvan consists primarily of residues connected by α-(1→4)- and β-(1→4)- linkages. The branch was situated at the O-2 position of rhamnose, while the sulfated group was positioned at the C-3 position of rhamnose (Lahaye et al. 2007, Thu et al. 2015; Tziveleka et al. 2019). Among them, the A_3s_ type ulvan consisted of glucuronic acid linked to rhamnose via 1→4 glycosidic bonds, with rhamnose further connected to glucuronic acid through 1→4 glycosidic bonds to constitute the main chain. Modification at the C-3 position of rhamnose involved the addition of a sulfate group, with potential branches occurring at the C-2 position. This structural configuration represented the predominant disaccharide unit in ulvan. Substitution glucuronic acid for iduronic acid was another B_3s_ type of ulvan. Physicochemical property analysis showed that ulvan was a semicrystalline polymer structure devoid of a triple helix configuration (Gao et al. 2020). The unique chemical composition of ulvan results in its disordered conformation, a characteristic that is heavily influenced by the species and growth environment of the green algae from which it is derived.

EPs have garnered increased research attention in recent years. The monosaccharide composition, sulfate positioning, and sugar chain structure of EPs are influenced by the species, seasonal variations, and environmental conditions. Consequently, it is very difficult to elucidate the precise structure of EPs. Limited research has been conducted on the detailed chemical structures of EPs (Kim et al. 2011). Earlier studies indicated that polysaccharides from *Enteromorpha* consist of various linked Rha and Xyl units, including α-(1→4)-, α-(1→3)-, α-(1→3,4)-, and α-(1→2,3,4)-linked Rha units as well as β-(1→4)- and β-(1→2,4)-linked Xyl units (Ray et al. 1995). Yu et al. (Yu et al. 2017) extracted the polysaccharides from *E. prolifera* and analyzed the structure by MS and NMR, revealing that the backbone chain consisted of D-GlcUAp-α-(1→4)-3-sulfate-_L_-Rhap-β-(1→4)-_D_-Xylp-β-(1→4)-3-sulfate-_L_-Rhap units. Qi et al. (Qi et al. 2012) extracted sulfated polysaccharides from *E.clathrata* (FEP) and the backbone of FEP was composed of (1→4) glycosidically linked β-_L_-arabinopyranose residues, with a partial sulfate group at the C-3 position. Generally, green algae polysaccharides are composed of glucuronic acid, rhamnose, xylose, galactose, and fucose. Specifically, rhamnose is linked by 1→2,4 and 1→4 glycosidic bonds; glucose is linked by 1→4; galactose is linked by 1→3 and 1→6; glucuronic acid and xylose are linked by 1→4 and are located at the termini, with xylose being partially sulfated at the O-2 position (Chattopadhyay et al. 2007). The structure of EPs exhibit significant variation, evident not only in their chemical composition but also in the arrangement of glycosidic bond connections, the distribution of sulfuric acid groups, and the presence and location of branch points. Table 2 provides an overview of the monosaccharide composition and structural features of different types of EPs. It can be seen that *Enteromorpha* is a heteropolysaccharide containing a variety of structural units, complex and diverse connection modes, and branched chains.

The study of the structure of green algae polysaccharide is helpful to strengthen the high-value development and utilization of green algae resources. However, The intricate and diverse nature of glycosidic bond compositions and linkages, coupled with the presence of sulfate groups at various positions and branching points, renders the investigation of the fine structure of green algae polysaccharides both difficult and challenging.

**Table 1:** The monosaccharide composition and structure of different types of green algae polysaccharides

Species	Molecular mass/ Da	Structural features	Monosaccharide composition	Ref
*Ulva pertusa*	23.6 × 104	8.3% sulfate	Rhamnose: Xylose: Arabinose: Galactose: Glucose = 5.5: 3.8: 0.7: 0.3: 0.3	(Gao et al. 2020)
*Ulva pertusa*	14.4 × 104	19.9% sulfate	Rhamnose: Xylose: Glucose: Glucuronic acid: Fucose = 31.3: 19.9: 6.7: 5.5: 0.5	(Wan et al. 2022)
*Ulva pertusa*	37.7 × 104	26.0% sulfate	Arabinose: Galactose: Glucuronic acid: Mannuronic acid = 0.80: 0.30: 1.80: 6.80	(Han et al. 2021)
*Ulva fasciata*	2.9 × 104	11.7% sulfate	Rhamnose: Xylose: Glucose = 17.1: 9.9: 10.7	(Shao et al. 2013)
*Ulva lactuca*	46.6 × 104	23.7% total sugar content	Glucose: Arabinose: Xylose: Mannose: Sorlose = 1.9: 0.5: 0.3: 6.7: 0.5	(He et al. 2016)
*Ulva armoricana*	14.0 × 104 ~ 50.0 × 104	14.3%~19.1% sulfate	Rhamnose: Galactose: Glucose: Xylose = 40.0: 6.7: 26.2: 4.4	(Hardouin et al. 2016)
*Enteromorpha prolifera*	0.4 × 104	9.0% sulfate	Mannose: Rhamnose: Glucuronic acid: Galacturonic acid: Glucose: Galactose = 0.6: 12.5: 30.6: 3.3: 1.7: 21.7	(Lin et al. 2020)
*Enteromorpha prolifera*	10.4 × 104	18.6% sulfate	Rhamnose: Xylose: Mannose: Galactose: Glucose = 3.6: 1.1: 0.2: 0.8: 0.3	(Tang et al. 2013)
*Enteromorpha prolifera*	1.1 × 104	95.8% total sugar content	Rhamnose: Glucose: Xylose = 3.6: 1.2: 1.0	(Zhou et al. 2020)
*Enteromorpha prolifera*	1.2 × 106	19.9% sulfate	Mannose: xylose: galactose: arabinose: glucuronic acid = 5.10: 2.80: 1.20: 0.30: 0.30	(Liu et al. 2022)
*Enteromorpha prolifera*	0.8 × 104	16.0% sulfate	Rhamnose: Glucuronic acid: Xylose = 1.00: 0.41: 0.12	(Jin et al. 2020)
*Enteromorpha prolifera*	4.4 × 104	12.3% sulfate	Rhamnose: Glucose: Xylose: Galactose = 6.8: 1.9: 0.8: 0.4	(Shi et al. 2017)

**Table 2:** Structural compositions of different species *Enteromorpha*

Species	Structural composition	Ref.
*E. prolifera*	D-GlcA-α-(1→4)-3-sulfate-_L_-Rha-β-(1→4)-_D_-Xyl-β-(1→4)-3-sulfate-_L_-Rha/(1→4)-β-_L_-rhamnose and (1→4)-linked xylose with sulfate groups linked on rhamnose at the C-3 position	(Jiao et al. 2009; Yu et al. 2017)
*E. clathrata*	(1→4)-linked β-_L_-Ara residues with partial sulfate groups at the C-3 position	(Qi et al. 2012)
*E. compressa*	(1→4)-and (1→2,4)-linked-Rha, (1→4)-linked Xyl, and (1→4)-and terminally linked-glucuronosyl residues/(1→4)-β-_L_-rhamnose and (1→4)-linked xylose with sulfate groups linked on rhamnose at the C-3 position	(Ray 2006; Jiao et al. 2009)
*E. intestinalis*	(1→4)-β-_L_-rhamnose and (1→4)-linked xylose with sulfate groups linked on rhamnose at the C-3 position	(Jiao et al. 2009)

### Extraction and purification of green algae polysaccharides

The distribution of green algae polysaccharides is predominantly in the interstitium and cell wall, with a minor presence in the cytoplasm. The water-soluble sulfated polysaccharides constitute 8–29% of the dry weight, and the yield of polysaccharides obtained varies depending on the extraction and purification methods employed. Currently, the most extensively studied method involves direct extraction of water-soluble polysaccharides using hot water (80–100 °C) (Chattopadhyay et al. 2007; de Carvalho et al. 2020), although enzyme-assisted extraction methods can also be utilized to enhance polysaccharide yield (Wahlström et al. 2020).

The hot water extraction method mainly uses water as a solvent to induce plasma wall separation of cells through thermal action. The intracellular or intercellular substances dissolved in water are exuded by diffusion. Pankiewicz et al. (Pankiewicz et al. 2016) extracted ulvan by stirring in hot water at 75–85 ℃ for 7 h with a solid to liquid of 1:30. The supernatant was concentrated after centrifugation, yielding a polysaccharide content of 16.23% after concentration of the supernatant post-centrifugation. Chen et al. (Chen et al. 2021a) extracted the ulvan by incubation in water at 90 ℃ for 3 h with the ratio of solid to liquid 1:20, resulting in the acquisition of 17.8 ± 0.6% of polysaccharide. The traditional hot water extraction method had the problems of long extraction time and diminished yield, with the elevated temperature leading to polysaccharides undergo the phenomenon of self-degradation. Therefore, in order to facilitate the subsequent research on polysaccharides, optimization of the hot water extraction process needs to be rationally optimized. Polysaccharides obtained through hot water extraction commonly harbor soluble impurities, necessitating the addition of alcohol for impurity removal and enhancement of polysaccharide purity. Xu et al. (Xu et al. 2015) extracted the EPs in hot water (90 ℃) for 4 h, obtaining 21.96% of polysaccharide after concentration and ethanol precipitation. Furthermore, it was suggested that the extraction process of green algae polysaccharides could be improved by adjusting the pH value of the extraction solution to improve the purity of the polysaccharide. For example, Glasson et al. (Glasson et al. 2017) extracted ulvan into 1 L of 0.05 M HCl for 1 h and then adjusted the pH value to 7 with 1 M NaOH. Finally, 8.1 ± 1.0% of polysaccharide was obtained. Song et al. (Song et al. 2010) treated the sample with 0.05 M HCl for 2 h and obtained 86.1% of EPs. The chemical extraction method is more efficient in terms of extraction time when compared to the hot water extraction method. However, it significantly raises the cost associated with the disposal of acid and alkali waste disposal.

The extraction methods for green algae polysaccharides have been continuously optimized and updated as research progresses. The physically assisted extraction method primarily involves the disruption of plant cell wall structure through physical mechanisms, facilitating the extraction of polysaccharides from the cells and thereby reducing extraction time and increasing the yield of polysaccharides. The ultrasonic extraction method used ultrasonic waves to disrupt the cell wall structure, facilitating the dissolution of polysaccharides and thereby shortening extraction time and improve the extraction efficiency. Guo et al. (Guo et al. 2010) extracted EP in 28 min using ultrasound and achieved an extraction of 25.84 mg/g of crude polysaccharide. Chen et al. (Chen et al. 2021a) obtained 20.6 ± 1.2% ulvan through ultrasonic treatment for 30 min followed by extraction in a water bath at 90 °C for 2.5 h with a material-liquid ratio of 1:20. However, the ultrasonic method exhibited variability in extraction efficiency despite its shorter extraction time. Microwave-assisted extraction could better maintain the biological activity of polysaccharides. Tsubaki et al. (Tsubaki et al. 2016) employed microwave-assisted technology to extract ulvan, achieving a polysaccharide yield of 40.4 ± 3.2%. The utilization of physical assistance in the extraction process has the potential to significantly enhance extraction efficiency. While microwave-assisted extraction offers the advantage of reduced extraction time, it may be susceptible to issues such as uneven heating.

The principle of enzyme-assisted extraction involves the enzymatic degradation of cell walls under mild reaction conditions. The specificity of enzymes allows for targeted substrate degradation while minimizing damage to non-targeted substances (Li et al. 2016a). This method can increase yield, decrease processing time and lower overall costs. In particular, enzymes such as cellulase and pectinase have been shown to effectively break down cell wall, leading to increased release of (Fernandes et al. 2019). Hardouin et al. (Hardouin et al. 2016) added six enzymes including protease and cellulase into *Ulva* extraction, and the polysaccharide yield reached 35.3 ± 0.3%. Lü et al. (Lu et al. 2014) introduced protease into the extraction solution and obtained an EP yield of 27.75%. These findings underscore the significant enhancement in polysaccharide yield that can be achieved through the efficient and mild action of enzymes.

In conclusion, there exist multiple extraction techniques for green algae polysaccharides, each presenting distinct advantages and limitations (Table 3). The amalgamation of different methods can greatly improve the efficiency of polysaccharide extraction. Chen et al. (Chen et al. 2021a) employed a combined approach of ultrasonic-assisted extraction and enzyme-assisted extraction to isolate ulvan, resulting in a yield of 26.7 ± 0.9%, surpassing that of individual methods such as hot water extraction, enzyme-assisted extraction, and ultrasonic-assisted extraction. This study suggests that the integration of diverse extraction methods can enhance the yield of the yield of green algae polysaccharides, reduce the production cost, and provide a basis for further research.

**Table 3:** Advantages and disadvantages of different extraction methods

Methods	Advantages	Disadvantages
Hot water extraction	A. Simple operation. B. Low cost and suitable for large-scale industrial extraction.	A. Long time. B. Low extraction rate. C. High temperatures may cause degradation of polysaccharides.
Alkali solution extraction	A. Save time.	A. May disrupt polysaccharide structure. B. Increased cost of waste liquid treatment.
Microwave assisted extraction	A. Save time and high extraction efficiency.	A. Can not improve polysaccharide yield and purity.
	B. Maintain the structure and biological activity of polysaccharide.	B. Uneven heating.
Ultrasonic assisted extraction	A. Save time.	A. The extraction efficiency is not stable.
	B. Improve the homogeneity of raw material and solvent mixing degree.	
Enzyme assisted extraction	A. Save time and high extraction efficiency.	A. Enzymes are easily inactivated.
	B. Low reaction temperature.	B. Enzymes are more expensive.

The crude polysaccharides obtained through the aforementioned method is found to contain proteins, small molecules, and other non-polysaccharide impurities, necessitating additional purification steps to ensure its suitability for subsequent structural analysis and investigation of biological activity. The protein can be removed by adding protease to the extract for hydrolysis and Savage method, followed by dialysis to remove small molecular impurities generated during hydrolysis (Lin et al. 2020). Further purification is performed through ion exchange chromatography (IEC) or gel permeation chromatography (GPC). The green algae polysaccharide exhibits varying charges at specific pH levels, allowing for the purification and separation of its components based on their charge differences. Glasson et al. (Glasson et al. 2022) used ion exchange chromatography with a Q Sepharose column and gradient elution to achieve purification, resulting in final polysaccharide yields of 1.45, 1.29 and 2.8%. Li et al. (Li et al. 2020a) utilized ion exchange chromatography with a DEAE-Sepharose column to purify ulvan from *U. pertusa*, successfully eluting three distinct polysaccharide components with 0 M, 0.5 M and 1 M NaCl. Pan et al. (Pan et al. 2019) used DEAE Sepharose Fast Flow with a NaCl concentration of 0.5-1 M for the purification of four polysaccharide components from *E. prolifera*. Chi et al. (2020a, 2021) used HiTrap Q FF gel for the purification of ulvan extracted from *U. clathrata* with a NaCl gradient of 0–2 M. Lv et al. (Lv et al. 2013) employed Sephadex G-100 gel with water as the mobile phase to isolate two EP components. In addition, Xu et al. (Xu et al. 2015) used the gel column SephacryTm S-300 h to isolate two components of EP. In summary, IEC relies on the different charges of polysaccharides to achieve the separation of different components of green algae polysaccharides, whereas GPC leverages the difference in molecular size of polysaccharides to achieve the separation of different components. Therefore, IEC may be more suitable for polysaccharide components with minimal disparity in molecular weight but different charges, whereas GPC may be more appropriate for components with similar charges but large differences in molecular weight (Table 4).

**Table 4:** Advantages and disadvantages of different purification methods

Methods	Advantages	Disadvantages
Dialysis	A. Simple operation. B. Save time. C. Reduce the loss of polysaccharides.	A. Incomplete purification. B. Requirements for molecular weight.
Ultrafiltration		
Ion exchange chromatography	A. High sensitivity. B. High separation efficiency. C. High reliability.	A. Low productivity. B. Strict sample conditions. C. Requires large amounts of buffer and eluent.
Gel permeation chromatography	A. High sample recovery.	A. Slower separation operation. B. The resolution is low, especially between molecules of similar relative molecular mass.
	B. Good repeatability of the experiment. C. No change in sample biological activity.	

**Fig. 6:**
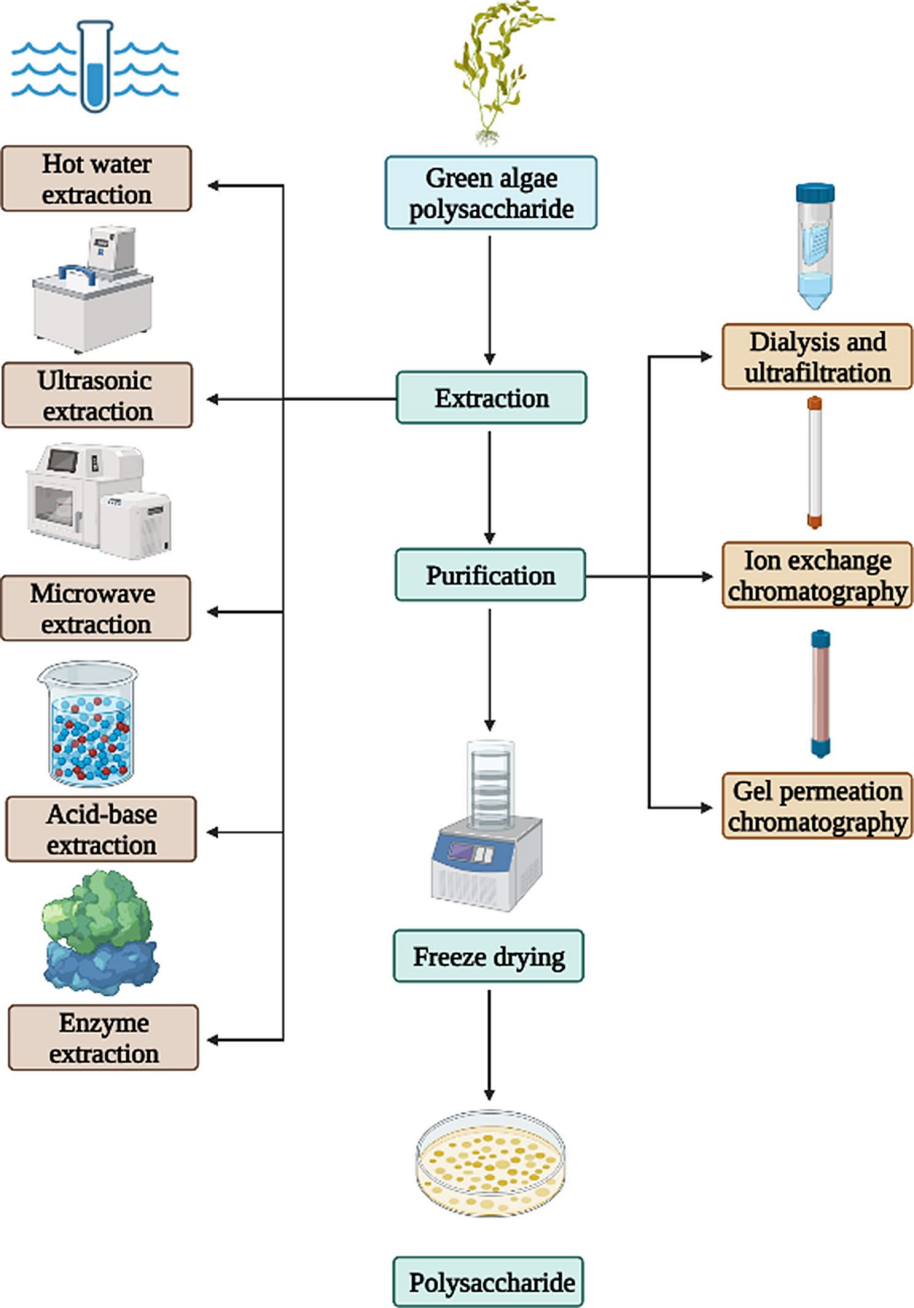
The main methods in green algae polysaccharides extraction and purification

The biological activities and application effects of green algae polysaccharides are intricately linked to their purity. Polysaccharides with high levels of purity exhibit enhanced biological activities, including antioxidant, immunomodulatory, anticoagulant, and antiviral properties, which play a significant role in the advancement of pharmaceuticals and health products. Nevertheless, the cost of commercial polysaccharides remains high due to the intricate purification process and low yield. Therefore, it is imperative to devise an appropriate method for the separation and purification of green algae polysaccharides. The process of extracting and purifying green algae polysaccharides is shown in Fig. 6.

### Activity of green algae polysaccharide

#### Antioxidant activity

When the body is in a state of oxidative stress, an abundance of free radicals accumulated and attack sugars, proteins, lipids and DNA within the body, leading to damage of cell membranes structures, oxidative harm to cells, and a decline in immune function. Oxidative stress can trigger a variety of related diseases, such as inflammation, cardiovascular disease, and tumors (Pisoschi et al. 2015, Huang et al. 2017). In vitro studies have shown antioxidant properties in polysaccharides extracted from green algae (Zhang et al. 2010). Green algae polysaccharides are an acidic polysaccharide containing sulfate. Higher sulfate content is associated with increased biological activity of this polysaccharide. Barakat et al.(Barakat et al. 2022) successfully extracted ulvan from *U. fasciata*, which contained 20.45% sulfate and demonstrated significant antioxidant activity, achieving 84.93% scavenging of DPPH radicals. Chen et al. (Chen et al. 2021a) employed enzyme-assisted extraction techniques to obtain ulvan, which exhibited superior and more pronounced DPPH free radical scavenging activity (SC 6.52 mg/mL) compared to ulvan extracted using hot water-assisted and ultrasound-assisted methods. It was observed that low molecular weight ulvan fractions possessed higher antioxidant capacities relative to their high molecular weight counterparts (Li et al. 2018b, c). Additionally, green algae polysaccharides can also significantly enhance the activity of endogenous antioxidant enzymes such as catalase (CAT), glutathione peroxidase (GSH-Px) and superoxide dismutase (SOD), while reducing levels of malondialdehyde (MDA). This mechanism may help alleviate oxidative stress caused by aging and hyperlipidemia (Xu et al. 2019; Li et al. 2020a; Yang et al. 2020). Wassie et al. (Wassie et al. 2022) demonstrated an increase in catalase (CAT) activity, total antioxidant capacity, and superoxide dismutase (SOD) activity, along with a decrease in malondialdehyde (MDA) levels in the serum of birds administered with EP.

This suggests that green algae polysaccharides have good free radical scavenging ability in vitro and affect the expression of antioxidant enzymes in vivo, thereby contributing to their antioxidant properties. However, the specific mechanism by which they modulate antioxidant molecular signaling pathways remain largely unexplored, necessitating further research in this area. In addition, the antioxidant capacity of these polysaccharides is dependent on their molecular weight, of polysaccharides, a topic that will be elaborated upon in the subsequent section on oligosaccharide activity (Qi et al. 2006).

#### Immune regulatory activity

Immunity serves as a defense mechanism for organisms against foreign pathogens, with numerous algae polysaccharides exhibiting immunomodulatory activity that can also regulate innate immune function. Specifically, green algae polysaccharides have been found to possess immunomodulatory activities that primarily involve the activation of immune responses and the modulation of immune cell activity (Zhao et al. 2016). Xu et al. (Xu et al. 2005) discovered that appropriate concentrations of EP had a notable impact on the proliferation of T and B lymphocytes, as well as on the increased production of interferon-γ (IFN-γ) triggered by the activation of antigen-presenting cells. Sulfated ulvan was found to regulate the levels of natural killers (NK) including tumor necrosis factor (TNF-α), interleukin-6 (IL-6), interleukin-1β (IL-1β), and interferon-c (IFN-c) (Meng et al. 2016; Wu et al. 2016). In *S. senegalensis* macrophages, ulvan has a stimulatory effect that is enhanced when delivered via nanoparticles (Fernández-Díaz et al. 2017). Furthermore, various molecular weight fractions (7、9、13、21 and 209 kDa) were derived from *U. ohnoi*, all of which exhibited immunomodulatory properties (Kidgell et al. 2020). An investigation into ulvan from *U. intestinalis* demonstrated that the immunomodulatory activity of the lower molecular weight fraction (28.7 kDa) was markedly superior to that of the higher molecular weight fraction (87.2 kDa) (Tabarsa et al. 2018). Additionally, treatment of the porcine intestinal epithelial cell line (IPEC-1) with purified low molecular weight (4.4 kDa) ulvan from *U. armoricana* (at concentrations ranging from 5 to 500 µg/ml) resulted in increased mRNA and protein expression levels of cytokines such as CCL20, IL-8, and TNF-α (Berri et al. 2017). These findings underscore the significance of interactions among the structural characteristics (e.g., molecular weight and degree of sulfation) of green algae polysaccharides in determining their biological activity. While a clear correlation exists between molecular weight, sulfation level, and the degree of immunomodulation exhibited by green algae polysaccharides, the intricate nature of their structural composition complicates the elucidation of the precise relationship between immunomodulation and specific structural features.

Hence, green algae polysaccharides can promote the production of cytokines and participate in the regulation of the body’s immune response, thereby serving as a potential immunomodulatory agent. Green algae polysaccharides can also be used as a nutritional supplement to enhance immune function.

#### Anticoagulant activity

The anticoagulant properties of seaweeds have been the subject of research for over six decades. Brown, red, and green algae all exhibit anticoagulant activity. The primary bioactive compounds responsible for this activity are various sulfated polysaccharides. Specifically, galactan sulfate and fucoidan sulfate are the active components in red and brown algae, respectively (Pozharitskaya et al. 2020; Ajarem et al. 2021; Li et al. 2021). In contrast, the anticoagulant activity in green algae is primarily attributed to arabinogalactan sulfate or rhamnogalactan sulfate (Wang et al. 2013). The intrinsic and extrinsic pathways culminate in the production of thrombin, which subsequently converts soluble fibrinogen into insoluble fibrin, leading to the formation of a blood clot. The mechanism of action of green algae polysaccharides primarily involves the enhancement of antithrombin III and heparin cofactor II, or the direct inhibition of thrombin activity and fibrin polymerization, both of which are critical endogenous inhibitors (Matsubara 2004; Adrien et al. 2017; Cui et al. 2018). Heparin is one of the main clinical drugs for the treatment and prevention of thrombosis, but it has some side effects such as bleeding, thrombosis syndrome and thrombocytopenia (Yu et al. 2018). The current research emphasis is on identifying alternative heparin analogs that can effectively prevent or treat cardiovascular diseases while possessing potent anticoagulant activity. Green algae polysaccharides have been identified as having anticoagulant effect, with its efficacy primarily assessed through measurements of thromboplastin time, thrombin time, and prothrombin time. It has been reported that the anticoagulant activity of green algae polysaccharides surpasses those of red algae and brown algae (Shanmugam et al. 2001; Athukorala et al. 2007).

The anticoagulant activity of green algae polysaccharides is typically associated with factors such as molecular size, monosaccharide composition, sulfate content, sulfate positioning, and attachment mode. For instance, ulvan derived from *U. linza* could extend the activated partial thromboplastin time (aPTT) by 3.3 to 6.2 times relative to the normal clotting time, contingent upon its degree of sulfation and molecular weight (Wang et al. 2013). Notably, lower concentrations of sulfated ulvan exhibit a prolonged anticoagulant effect compared to the commercially available anticoagulant Lovenox (Adrien et al. 2019). Cui et al. (Cui et al. 2018) conducted an extraction of polysaccharides from *E. linza*, subsequently obtaining five low molecular weight fractions through enzymatic hydrolysis. Their findings indicated a positive correlation between the degree of sulfation and anticoagulant activity, with the latter being retained until a molecular weight threshold of less than 200 kDa was reached. Mao et al. (Mao et al. 2006) studied polysaccharides from *U. Conglobata* and showed that the polysaccharide could prolong thrombin times which inhibited thrombin and modulated heparin cofactor II. Mao (Mao et al. 2009) isolated polysaccharides from *Monostroma. Latissimum* and revealed that its heightened anticoagulant activity was attributed to its high rhamnose content. Therefore, the findings suggest that green algae polysaccharides have the potential to be used as a novel anticoagulant agent. The anticoagulant activity of the sulfated polysaccharides was attributed, in part, to the strong interactions between the negatively charged sulfate esters and certain positively charged peptidic sequences of proteins involved in the coagulation process. Nevertheless, there is a paucity of research on the effect of monosaccharide composition on bioactivity, warranting further investigation.

#### Antiviral activities

Green algae polysaccharides are increasingly recognized as a new source of antiviral activity within the realm of natural compounds (Komatsu et al. 2013). Research has demonstrated that green algae polysaccharides exhibit inhibitory properties against many viruses, such as herpes simplex virus, cytomegalovirus, human immunodeficiency virus, and influenza virus. Lee et al. (Lee et al. 1999) successfully isolated sulfated rhamnosan from *M. latissimum* which displayed significant inhibitory effects on virus replication. Shefer S et al. (Shefer et al. 2021) evaluated the anti-SARS-CoV-2 activity of ulvan extracted using ammonium oxalate and hydrochloric acid. The results indicated that the AOX protocol was able to protect VERO E6 cells from SARS-CoV-2 cytopathy, with an inhibitory effect 11.3 times greater than that of the HCl protocol. These findings indicate that the more negatively charged sulfate groups facilitates interactions with viral envelope glycoproteins or surface receptors, leading to a reduction in viral entry into host cells. In vitro studies of ALV-J, ulvan have been found to effectively bind to viral particles, thereby preventing ALV-J from adsorbing to host cells and causing a notable decrease in the expression of ALV-J gene and gp85 protein (Sun et al. 2018). Furthermore, EPs have been shown to hinder the adsorption and penetration of herpes simplex virus (HSV) into laryngeal epithelial cancer cells, as well as inhibit HSV replication and transcription (Lopes et al. 2017). The potential antiviral natural active substances of green algae polysaccharides, particularly ulvan, have been extensively studied. Given the paucity of research on the antiviral properties of green algae polysaccharides, the comprehension of their conformational relationships remains constrained. Nonetheless, the conformational characteristics of green algae polysaccharides exhibiting antiviral activity appear to be analogous to those observed in other sulfated polysaccharides (Witvrouw et al. 1997, Ghosh et al. 2009).

#### Hypolipidemic activity

Hyperlipidemia is associated with cerebrovascular and cardiovascular complications, as well as atherosclerosis, a prevalent endocrine disorder (Song et al. 2017, Chen et al. 2018; Zhao et al. 2019). In recent years, reports have confirmed the hypolipidemic effects of green algae polysaccharides. In a hyperlipidemia mouse model, ulvan significantly decreased serum total cholesterol (TC), triglycerides (TG), and low-density lipoprotein cholesterol (LDL-C) levels, while increasing high-density lipoprotein cholesterol (HDL-C) levels, suggesting its potential therapeutic efficacy (Li et al. 2020a). The molecular weight and degree of sulfation of green algae polysaccharides influence their hypolipidemic activity. Peng et al. (Pengzhan et al. 2003) incorporated two distinct molecular weights of ulvan (151.6 kDa and 28.2 kDa) into the diets of rats subjected to a high-cholesterol regimen to evaluate their antihyperlipidemic effects. The findings indicated that ulvan with a higher molecular weight significantly reduced serum total cholesterol and low-density lipoprotein (LDL) cholesterol levels, whereas the lower molecular weight ulvan fraction was more effective in decreasing triglycerides and increasing high-density lipoprotein (HDL) cholesterol levels. Teng et al. (Teng et al. 2013) also investigated which demonstrated that EPs presented high anti-hypolipidemic activities by suppressing body weight gain and reducing levels of TG, TC, and LDL-C levels in both plasma and liver. Furthermore, EPs were found to possess pancreatic lipase inhibition activities (Yuan et al. 2018). The hypolipidemic activity of green algae polysaccharides may be achieved by a combination of multiple mechanisms. However, there are few studies available on the specific antilipidemic mechanism, indicating a necessity for further investigation in this area.

## Green algae oligosaccharide

### Preparation of green algae oligosaccharide

The utilization of green algae polysaccharides in the food and pharmaceutical industries is constrained by its limited solubility and low bioavailability, as it is the main constituent of green algae. Green algae oligosaccharides, a degradation product of polysaccharides, have attracted more and more attentions due to its retention of various activities exhibited by polysaccharides, as well as its enhanced solubility and bioavailability (Liu et al. 2019). The preparation of green algae oligosaccharides can be categorized into three categories: chemical degradation, physical degradation, and enzymatic degradation. Yu et al. (Pengzhan et al. 2003) utilized microwave thermal degradation of ulvan to prepare *Ulva* oligosaccharides, resulting in the preservation of glycosidic linkages without destroying the important structural units. In a separate study, Zhang et al. (Zhang et al. 2013) employed ascorbic acid and H_2_O_2_ to degrade the EPs and obtain oligosaccharides. The limited studies on the degradation of polysaccharides by chemical and physical methods are attributed to the energy-intensive nature of physical degradation which necessitates significant energy input to break the glycosidic bonds, and the use of strong acids or strong oxidizing properties in chemical degradation to break glycosidic bonds.

Both methods suffer from long reaction times and difficult to control reaction conditions. However, enzymatic hydrolysis has attracted wide attention because of its mild reaction conditions and precise product specificity. Currently, the primary enzymes used in the degradation of ulvan are PL24, PL25, PL28 and PL40 ulvan lyases, which are specialized enzymes designed for ulvan degradation (Table 5). Ulvan lyases act by cleaving the β-(1→4)-glycosidic bond between Rha3S and GluA or IduA primarily by a β-elimination mechanism, resulting in the production of oligosaccharides with 2–4 degrees of polymerization (Dps) (Ulaganathan et al. 2017, 2018a, 2018b). This is the rationale behind the formation of even-odd oligosaccharides of DP2 and DP4 as the degradation products of most ulvan lyases targeting ulvan. The β-elimination mechanism effectively maintains the unique sugar structures present in ulvan, thereby facilitating the potential for valuable advancements in ulvan utilization. Despite this, there is a lack of literature documenting specific enzymes capable of degrading EPs. Only Li et al. (Li et al. 2013, 2016b, c) isolated *Alteromonas* sp. A321 as a novel EP-degrading strain, although the exact classification of the enzymes responsible as either polysaccharide lyases or glycoside hydrolases remains uncertain. Zhang et al. (Zhang et al. 2016) used enzymes that produced by *Alteromonas* sp. A321 to degrade the EPs and obtained 61.21% of the oligosaccharides. Therefore, it is also a problem worth further discussion whether there is a special degradation EPs enzyme.

**Table 5:** The products of ulvan lyase from different sources

Sources	PL family	Mw (kDa)	Products	Product Composition	Ref.
*Alteromonas* sp. KUL17	PL24	55(110.85)	DP2, 4, 6	-	(He et al. 2017)
*Alteromonas* sp. LOR_107	PL24	59.64	DP2, 4	Δ-R3S, Δ-R3S-IdoA-R3S, Δ-R3S-Xyl-R3S	(Kopel et al. 2016)
*Pseudoalteromonas* sp. PLSV_3875	PL24	59.62	DP2, 4	ΔUA-R3S, ΔUA-R3S-IdoA-R3S	(Qin et al. 2018)
*Pseudoalteromonas* sp. PLSV_3925	PL24	111.4	DP2, 4	ΔUA-R3S, ΔUA-R3S-IdoA-R3S	(He et al. 2017)
*Vibrio* sp. FNV38	PL24	54	DP2, 4	Δ-Rha3S, Δ-Rha3S-HexA-Rha3S, Δ-Rha3S-Xyl-Rha3S	(Rodrigues et al. 2024)
*Alteromonas* sp. LOR_29	PL25	52	DP2, 4	Δ-R3S, Δ-R3S-Xyl-R3S	(Foran et al. 2017)
*Pseudoalteromonas* sp. PLSV_3936	PL25	54.28	DP2, 4	ΔUA-Rha3S, ΔUA-Rha3S-Xyl-Rha3S	(Ulaganathan et al. 2017)
*Alteromonas* sp. A321. ALT3695	PL25	53	DP2, 4	∆GlcA-Rha3S, ∆GlcA-Rha3S-Xyl-Rha3S	(Gao et al. 2019)
*Thalassomonas* sp. LD5	PL25	54.54	DP2, 4	∆Rha3S, ∆Rha3S-Xyl-Rha3S	(Wang et al. 2022)
*Alteromonas* sp. TK-45 (2)	PL25	51.99	DP2, 4	∆GlcA-Rha3S, ∆GlcA-Rha3S-Xyl-Rha3S	(Tang et al. 2022)
*Alteromonas* sp. KUL_42	PL25	53.97	DP2-4	Rha3S-GlcA, Rha3S-Xyl-Rha, Rha3S-lduA-Rha, Rha3S-lduA-Rha3S-Xyl, Rha3S-lduA-Rha3S-Xyl2S	(Li et al. 2023)
*Alteromonas* sp. 76–1	PL25	54.39	DP2-4	∆Rha3S, Rha3S-GIcA-Rha, ∆Rha3S-IduA-Rha3S, ∆Rha3S-Xyl-Rha3S	(Tang et al. 2023)
*Nonlabens Ulvanivorans* NLR42	PL28	46	DP2, 4	∆-R3S, ∆-R3S-Xyl-R3S	(Collén et al. 2011)
*Formosa agariphila* KMM 3901^T^	PL28	54.73	DP2,4	Δ-Rha3S, Δ-Rha3S-GlcA-Rha3S, Δ-Rha3S-IdoA-Rha3S	(Reisky et al. 2018)
*Tamlana fucoidanivorans* CW2-9	PL28	52	DP2,4	ΔRha3S, ΔRha3S-IduA-Rha3S, ΔRha3S-GlcA-Rha3S	(Xu et al. 2024)

### Activity of green algae oligosaccharide

At present, there is a growing body of literature on the activity of green algae oligosaccharides. However, these studies are fragmented. Moreover, the intricate structure of oligosaccharides has hindered the determination of the mechanism of their activity and the structure-activity relationship. Li et al. (Li et al. 2020b) studied the anti-inflammatory effects of *Ulva* oligosaccharides on bowel disease (IBD). The results indicated that a dosage of 50 mg/kg of *Ulva* oligosaccharides exhibited a protective effect on IBD, with the best protective effect observed at concentration between 100 and 120 mg/kg. Carvalho et al. (de Carvalho et al. 2020) found that the existence of *Ulva* oligosaccharide could improve the anti-cancer activity of ulvan. Liu et al. (Liu et al. 2010) observed that *Enteromorpha* oligosaccharides could significantly stimulate the secretion of nitric oxide (NO), upregulate the expression of cytokines such as IL-1β, IL-6 and TNF-α, and activate inflammatory agents such as iNOS, COX2, and NLRP3, thereby activating the immune system. In addition, Tabarsa et al. (Tabarsa et al. 2018) discovered that *Ulva* oligosaccharides containing low molecular weight and high sulfate group components could induce proliferation of RAW264.7 macrophages and prompt the release of significant amounts of nitric oxide, IL-1β, TNF-α, IL-6, IL-10, and IL-12 cytokines from RAW264.7 cells, suggesting a mild immunomodulatory activity that is closely related to molecular weight. In a related study, Qi et al. (Qi et al. 2006) prepared *Ulva* oligosaccharides with different molecular weights, and found that the degraded form exhibited greater antioxidant activity compared to the higher molecular weight ulvan. Wang et al. (Wang et al. 2013) discovered that *Enteromorpha* oligosaccharides possessed an anticoagulant activity which depended on their degree of acidification, the molecular and the distribution of sulfuric acid groups. Yu et al. (Pengzhan et al. 2003) studied the anti-hyperlipidemic activity of *Ulva* oligosaccharides in male Wistar rat models, revealing that *Ulva* oligosaccharides exhibited greater efficacy compared to polysaccharides in managing hyperlipidemia associated with diabetes. All these results indicate that low molecular weight green algae oligosaccharides possess superior bioavailability. Given the comparable activity of oligosaccharides to natural polysaccharides, they present a more advantageous option for the formulation of dietary supplements and pharmaceuticals.

To sum up, due to the complexity of the structure, the active mechanism of green algae oligosaccharides is not clear, but it can be hypothesized that it is related to some active groups produced by the degradation of polysaccharides. At present, researches on the activity of green algae oligosaccharides are relatively shallow, and there is no appropriate method to obtain oligosaccharide with a refined structure.

## Conclusion and perspective

In this review, the progress of research on the composition, structure and biological activity of green algae polysaccharides have been investigated and summarized. Recent studies have proved the significant impact of green algae polysaccharides on human health and nutrition, showcasing their various physiological activities such as immune regulation, anticoagulant effects, and hypolipidemic activities. These attributes make green algae polysaccharides a promising candidate for the treatment of conditions including hyperlipidemia, hypertension, and other metabolic diseases, positioning them as an important source for the development of novel marine-based pharmaceuticals.

**Fig. 7:**
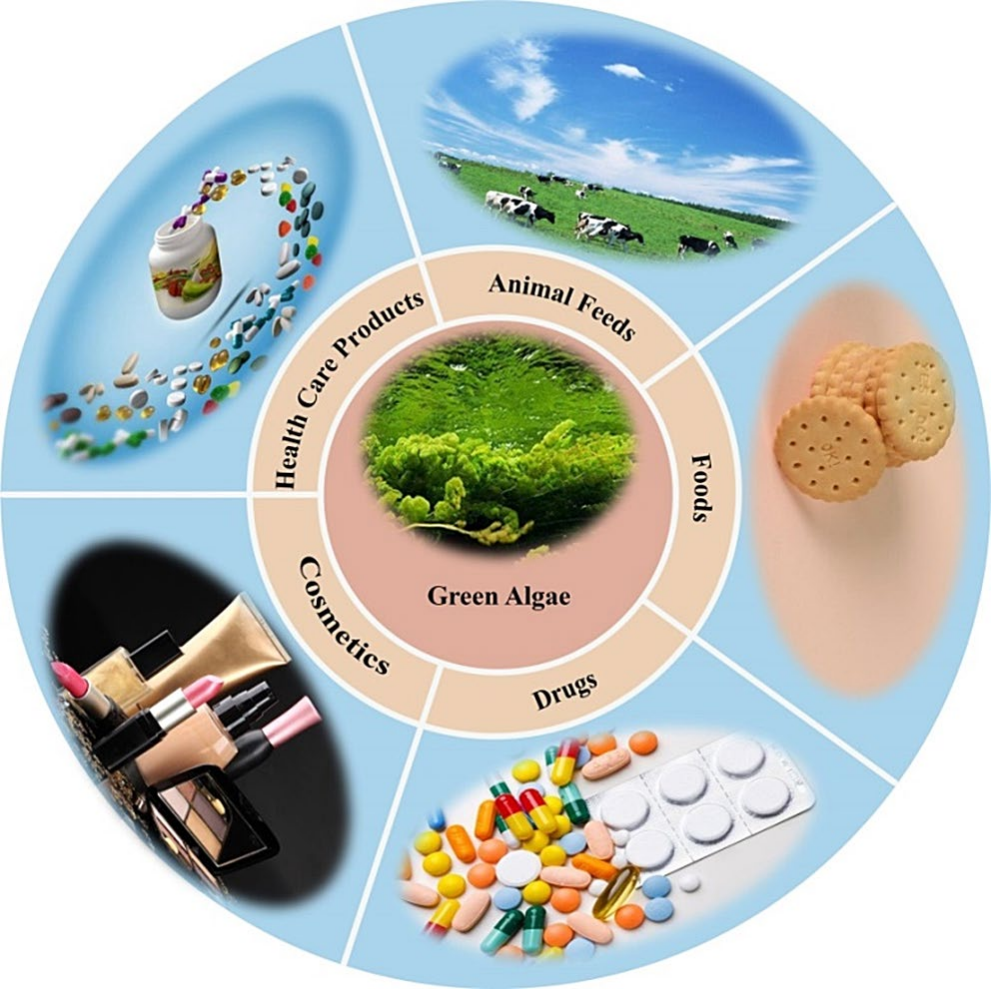
Potential application prospects of green algae polysaccharides

The green algae polysaccharides also can eliminate the oxidative radicals such as DPPH, OH^−^ and O^2−^. The utilization of these biological activities in the development of new functional foods and pharmaceuticals for the management of diet-related chronic disorders holds promise. However, the exploration and utilization of green algae polysaccharides face numerous challenges due to its intricate structure, susceptibility to various influencing factors, high biological molecular weight, and low bioavailability. For instance, Ji et al. [103] isolated polysaccharides from *E. clathrata* at different harvesting times, demonstrating variations in the ratios of these polysaccharides. Moreover, while crude polysaccharides are easily extracted, their low purity hinders effective utilization. Thus, the purification of large-scale refined polysaccharides remains a key research focus. In conclusion, the myriad advantageous properties of green algae polysaccharides render them highly promising for applications in the realms of food, cosmetics, and biomedicine, thereby garnering considerable attention (Fig. 7). The escalating interest in green algae polysaccharides within the marine bioresources sector underscores their emergence as a focal point of discussion, accompanied by both opportunities and challenges coexist.

## Data Availability

Not applicable.
